# Morbidity and radiographic outcomes of severe scoliosis of 90° or more: a comparison of hybrid with total pedicle screw instrumentation

**DOI:** 10.1007/s11832-014-0604-1

**Published:** 2014-08-02

**Authors:** Ilkka Helenius, Mikko Mattila, Tuomas Jalanko

**Affiliations:** 1 Department of Paediatric Orthopaedic Surgery, Turku University Central Hospital, Kiinamyllynkatu 4-8, 21520 Turku, Finland; 2 Hospital for Children and Adolescents, Helsinki University Central Hospital, Helsinki, Finland

**Keywords:** Severe scoliosis, Hybrid instrumentation, Pedicle screw instrumentation, Mortality

## Abstract

**Objectives:**

Untreated severe scoliosis is associated with increased mortality and remains a significant surgical challenge. Few studies have reported mortality after the surgical treatment of severe scoliosis beyond a 2-year follow-up. The objectives of this study were to evaluate mortality beyond standard 2-year follow-up and compare radiographic outcomes using hybrid or pedicle screw instrumentation for severe scoliosis.

**Methods:**

We evaluated 32 consecutive patients [11 males, mean age at surgery 15.3 (range 10.7–20.7) years] operated for a scoliosis of 90° or more using either hybrid (*n* = 15) or pedicle screw (*n* = 17) instrumentation. The follow-up time averaged 2.9 (2.0–6.6) years for radiographic and quality of life measurements and 5.5 years (2.0–9.0) years for mortality data. Of these patients, one had adolescent idiopathic scoliosis, three secondary scoliosis, and 28 neuromuscular scoliosis. Twelve patients in the hybrid and two patients in the pedicle screw groups underwent anteroposterior surgery (*p* < 0.001), and three patients in both groups had an apical vertebral column resection.

**Results:**

One (3.1 %) patient died during follow-up for severe pneumonia. Preoperatively, the mean magnitude of the major curve was 109° (90°–127°) in the hybrid and 100° (90°–116°) in the pedicle screw groups (*p* = 0.015), and was corrected to 45° (19°–69°) in the hybrid and 27° (18°–40°) in the pedicle screw groups at the 2-year follow-up (*p* < 0.001), with a mean correction of the major curve of 59 % (37–81 %) in the hybrid versus 73 % (60–81 %) in the pedicle screw groups, respectively (*p* = 0.0023). There were six postoperative complications, including one transient spinal cord deficit necessitating reoperation in the hybrid group as compared with five complications in the pedicle screw group (*p* = 0.53).

**Conclusions:**

The mid-term mortality rate after the surgical treatment of severe scoliosis was low. Severe scoliosis can be treated safely with significantly better correction of the spinal deformity using pedicle screws than hybrid instrumentation.

## Introduction

Untreated severe scoliosis of 70° or more is associated with increased mortality as compared with the normal population [1]. Most patients with scoliosis of 60° or more present with major spinal deformity, restrictive lung disease, and, if left untreated, rapid progression of the deformity [2–4].

Surgical options to correct severe spinal deformities include anterior release and posterior instrumentation, pre- and perioperative halo-gravity traction, and spinal osteotomies, such as vertebral column resection (VCR) [3, 5–14].

Pedicle screw instrumentation (PSI) provides better biomechanical fixation than hybrid instrumentation using hooks, wires, and lumbar pedicle screws [15]. PSI has been shown to obtain better correction of severe scoliosis of 100° or more than using wires or hooks [3]. Instrumenting severe scoliosis of 90° or more involves small or even absent apical concave pedicles and the need for an in–out–in pedicle screw insertion technique [6]. On the other hand, the need for anteroposterior surgery seems lower due to the enhanced correction potential [3, 5–8]. In patients with over 100° of scoliosis, all posterior PSI with perioperative halo-femoral traction has been reported to obtain 44 % coronal correction of the spinal deformity [8].

A limited number of studies comparing different spinal instrumentation techniques for severe scoliosis of 90° or more exists [3, 5, 7]. Additionally, some of these studies are purely register-based or multisurgeon, thus further decreasing the information available on the details of surgical techniques applied as well as complications after a 2-year follow-up.

It is unclear whether spinal deformity surgery changes the life expectancy of severe scoliosis patients, who often present with basic neurologic condition. Mortality beyond a 2-year follow-up is rarely reported [16]. Thus, in addition to radiographic measurements, we gathered mortality data beyond the standard 2-year follow-up.

## Methods

### Patients

We identified all patients who underwent surgical correction for severe scoliosis of 90° or more between 2003 and 2011 with a minimum of 2 years radiographic follow-up at our hospitals. There were a total of 32 consecutive patients. The first 15 consecutive patients were operated using a hybrid instrumentation between 2003 and 2006, and the following 17 patients were operated using a PSI between 2006 and 2011 (Table 1). The radiographic follow-up rate was 100 % for both groups at the 2-year follow-up. Mortality data were gathered from the Finnish Official Cause of Death statistics up to a mean of 5.0 years (range 2.0–9.0 years) follow-up on all patients.

**Table 1:** Clinical characteristics of the study groups

Characteristic	Hybrid (*n* = 15)	Pedicle screw (*n* = 17)	Significance
Age at surgery (years)	15.9 (3.0)	14.8 (2.8)	0.31
Gender (male/female)	5/10	6/11	0.91
Radiographic follow-up (years)	2.9 (1.4)	2.9 (1.4)	0.93
Follow-up for mortality (years)	6.9 (2.3)	3.7 (1.4)	<0.001
Ambulatory (*n*)	2	2	0.89
Neuromuscular scoliosis	12	16	0.23
CP	9	7	
Syndromic	3	6	
MMC	–	1	
Myopathy	–	1	
Hereditary polyneuropathy	–	1	
Secondary scoliosis	2	1	
Adolescent idiopathic	1	0	

*CP* cerebral palsy, *MMC* myelomeningocele

### Study design

The study design was a retrospective study of prospectively collected data using two consecutive patient cohorts. Clinical medical records and radiographs of the spin were evaluated. Examinations were performed by one of four orthopedic spine surgeons before surgery, on the day when the patient was discharged from the hospital, 6 months, and at 2 years after surgery. All patients were operated by at least two out of three orthopedic spine surgeons, of which one was always the senior author. Pelvic fixation was performed when L5 tilt over the S1 endplate was over 10°. Standing or sitting posteroanterior and side radiographs of the whole spine (scoliosis) were taken preoperatively, immediately after surgery on the table, and during the ward period, as well as at 6 and 24 months. Two independent observers measured all radiographs. Spinal radiographs in case of difficult deformity were measured based on a consensus decision and, in borderline cases, the milder option was chosen. Standard preoperative imaging included anteroposterior spinal radiograph under traction and a full medical evaluation by a consultant pediatrician. Also, renal and cardiovascular systems were investigated using ultrasound for associated anomalies. Mortality data were collected on all patients from our National Official Cause of Death statistics. Data on complications were recorded in a prospective manner. The study was carried out from August 2003 to February 2013.

### Operative techniques

Anteroposterior surgery was performed in the hybrid instrumentation group when the thoracic curve magnitude exceeded 70°, thoracolumbar curves 100°, or in the immature patient to prevent crankshaft phenomenon. Anteroposterior surgery in the PSI group was performed in extreme curves of 100° with less than 25 % correction on traction films and in none for the prevention of crankshaft phenomenon. Anteroposterior surgery was performed in 12 (80 %) patients of the hybrid group (nine of them staged surgeries) and in two (12 %) patients of the pedicle screw group (*p* < 0.001). Briefly, the anterior approach was thoracotomy for the lower thoracic spine and thoracoabdominal for the Th12-L2 area. The patient is laid in a lateral decubitus position. Segmental vessels in the anterior fusion area were ligated unilaterally to allow wide and safe exposure of the spine, including anterior longitudinal ligament. Multilevel discectomies were performed for release of the rigid spinal deformity. Anterior spinal fusion was obtained through autologous rib grafting of the disc spaces. Then, the patient is turned prone and posterior elements of the spine are exposed carefully with electrocautery. Five (33 %) patients in the hybrid and 16 (94 %) patients in the pedicle screw groups underwent the Ponte procedure [17], consisting of wide facetectomies with resections of the spinous process (pedicle to pedicle), ligamentum flavum, and inferior and superior laminar borders (*p* < 0.001). Apical vertebral column resection (VCR) was performed for three patients in both groups. Patients in the hybrid group underwent VCR using a combined anteroposterior approach and patients in the pedicle screw group underwent VCR using an all-posterior approach according to previously described techniques [7]. All patients received morselized allogenic bone grafting when iliac fixation was performed.

Hybrid instrumentation included upper thoracic hook claw on both sides, sublaminar wires on the concave side, and midthoracic hooks on the convex thoracic spine and lumbar pedicle screws.

Pedicle screws were inserted with the free-hand technique based on posterior bony elements according to Kim et al. [18]. Multiaxial reduction screws are used at the apical concave side. Iliac fixation was used for six patients in the hybrid and 14 patients in the pedicle screw group using long iliac screws and connectors (*p* = 0.014).

Epidural bleeding is controlled using bipolar cauterization and the use of hemostatic agents such as human thrombin with gelatine matrix (FloSeal, Baxter US, Deerfield, IL). Spinal deformity correction is obtained by the double-rod cantilever maneuver or concave rod derotation.

Spinal cord monitoring [motor evoked potentials (MEPs), somatosensory evoked potentials (SSEPs), lumbar nerve root electromyography (EMG)] was performed in all of the operations. All patients were carefully followed up at the pediatric intensive care unit with a mean arterial pressure of 70 mmHg or more during the first 24 h. Lower leg movement was checked every 4 h. All patients were mobilized within the first postoperative week. Patients undergoing VCR were immobilized using thoracolumbosacral orthosis for 4 months postoperatively.

### Radiographic evaluation

Standard sitting or standing posteroanterior and lateral radiographs were taken of the entire spine pre- and postoperatively, and at follow-up visits. The proximal thoracic, main thoracic, and thoracolumbar/lumbar curves, and pelvic obliquity were measured from the posteroanterior radiographs and thoracic kyphosis (T5–T12), lumbar lordosis (T12–S1), and segmental kyphosis or lordosis were measured from the lateral radiographs [19, 20]. Coronal balance was determined as the horizontal distance of the spinous process of C7 from the center sacral line measured in millimeters. Sagittal balance was not evaluated radiographically, since most of the patients were nonambulatory.

### Statistical analysis

Values are given as means, standard deviations (SDs), or ranges. A two-tailed independent *t*-test was used to calculate the level of significance for continuous variables and the χ^2^-test was used for categorical variables. *p*-Values equal to or below 0.05 were considered statistically significant.

### Ethical aspects

We obtained permission to perform this study from the Ethics Committee of our University Hospital (ETMK 39/180/2011), where the study was conducted. All subjects gave Informed consent to participate in the study.

## Results

There were no deaths related to surgery. One of the patients died during the follow-up period. This death resulted from radiographically confirmed pneumonia 4 years and 10 months after spinal deformity surgery using pedicle screws.

### Radiographic outcomes

**Fig. 1:**
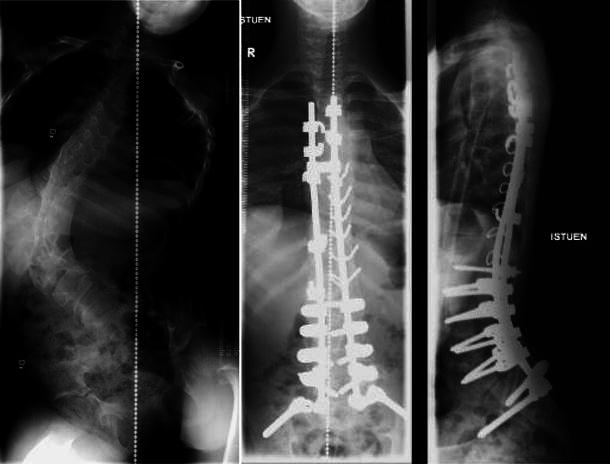
14-year-old boy with cerebral palsy (CP; spastic tetraparesis) and severe neuromuscular scoliosis. Anteroposterior spinal deformity correction with hybrid instrumentation provided excellent and stable correction at the 2-year follow-up

The mean preoperative Cobb angle of the major curve was 109° (range 90°–127°) in the hybrid group and 100° (90–116°) in the pedicle screw group (*p* = 0.015) (Table 1). The preoperative flexibility of the major curves in traction radiographs was similar in the study groups (29 ± 12 % in the hybrid vs. 33 ± 14 % in the pedicle screw group). The magnitude and correction of the major curve was significantly better in the pedicle screw group as compared with the hybrid group at the immediate postoperative (*p* = 0.0010 and *p* = 0.0044), 6 months (*p* < 0.001 and *p* = 0.0017), and 2-year postoperative radiographs (*p* < 0.001 and *p* = 0.0023) (Table 2). The average major curve corrections at 2 years were 59 % (range 37–81 %) for the hybrid and 73 % (range 60–81 %) for the pedicle screw groups, respectively (*p* = 0.0023) (Fig. 1).

**Table 2:** Comparison of radiographic data

	Hybrid (*n* = 15)	Pedicle screw (*n* = 17)	Significance
Major curve			
Preoperative (º)	109 ± 11	100 ± 8	0.015
Major curve on traction film (º)	76 ± 15	67 ± 15	0.13
Correction on traction (%)	29 ± 12	33 ± 14	0.43
Immediate postoperative (º)	48 ± 14	32 ± 11	0.0010
Correction (%)	56 ± 12	68 ± 10	0.0044
Postoperative 6 months (º)	43 ± 13	27 ± 11	<0.001
Correction (%)	61 ± 11	73 ± 9	0.0017
Postoperative 2 years (º)	45 ± 16	27 ± 8	<0.001
Correction (%)	59 ± 14	73 ± 7	0.0023
T5–T12 kyphosis			
Preoperative (º)	46 ± 16	39 ± 33	0.60
Immediate postoperative (º)	29 ± 15	29 ± 9.4	0.90
Postoperative 2 years (º)	33 ± 12	23 ± 9.6	0.034
T12–S1 lordosis			
Preoperative (º)	63 ± 17	57 ± 15	0.54
Immediate postoperative (º)	46 ± 12	59 ± 9	0.013
Postoperative 2 years (º)	49 ± 14	56 ± 9	0.19
Coronal balance (mm)			
Preoperative	44 ± 43	57 ± 26	0.41
Postoperative 2 years (º)	23 ± 19	23 ± 21	0.46
Pelvic obliquity (º)			
Preoperative	22 ± 16	23 ± 12	0.95
Postoperative 2 years	7 ± 6	6 ± 7	0.70

Values are expressed as mean ± standard deviation

Two patients in the hybrid and three in the pedicle screw groups had preoperatively thoracic hyperkyphosis (>40° kyphosis between T5 and T12) (Table 2). The mean thoracic kyphosis was significantly higher in the hybrid group than in the pedicle screw group at the 2-year follow-up (*p* = 0.034), and lumbar lordosis significantly lower in the hybrid than in the pedicle screw groups at the immediate postoperative radiographs (*p* = 0.013). There were no statistically significant differences of coronal balance or pelvic obliquity pre- and postoperatively between the study groups (Table 2). There was no radiographic evidence of a nonunion at follow-up.

### Clinical findings

The mean age at operation was 15.9 years (range 10.6–20.7) in the hybrid group and 14.8 years (range 9.6–19.6) in the pedicle screw group (*p* = 0.31) (Table 1). There were five boys in the hybrid group and six boys in the pedicle screw group. The mean operative times were 8 h 3 min (range 3 h 48 min to 12 h 30 min) in the hybrid group and 6 h 24 min (range 4 h 30 min to 8 h 15 min) in the pedicle screw group (*p* = 0.015), and the mean intraoperative blood loss was 4,300 ml (range 350–12,000 ml) and 2,700 ml (500-8,000 ml), respectively (*p* = 0.14) (Table 3).

**Table 3:** Comparison of perioperative data

Characteristic	Hybrid (*n* = 15)	Pedicle screw (*n* = 17)	Significance
Fused vertebral segments (*n*)	14.3 ± 0.96	15.0 ± 1.3	0.074
Iliac fixation (*n*)	6	14	0.014
Number of fixation points per fused segments	1.52 ± 0.059	2.0 ± 0.097	<0.001
VCR (*n*)	3	3	0.86
Operative time (h)	8.1 ± 2.1	6.4 ± 1.3	0.015
Anteroposterior surgery (*n*)	12	2	<0.001
Intraoperative blood loss (ml)	4,300 ± 3,350	2,700 ± 1,890	0.14

The mean implant density per fused vertebral bodies was significantly higher in the pedicle screw group than in the hybrid group (2.0 vs. 1.52; *p* < 0.001) (Table 3). Iliac fixation was carried out more often in the pedicle screw group (*p* = 0.014). There tended to be more fused segments in the pedicle screw group (15.0) as compared with the hybrid group (14.3) (*p* = 0.074). All ambulatory patients maintained their walking ability.

### Complications

The overall complication rates were rather similar between the hybrid [40 %, (6/15)] and the pedicle screw groups [29 %, (5/17)] (*p* = 0.53) (Table 4). The complications in the hybrid group were: one pneumothorax due to central venous access treated with pleural drainage, one prolonged pneumonia needing intensive care and ventilator treatment, one superior mesenterial artery syndrome necessitating total parenteral nutrition, one implant failure, one urinary retention and urosepsis, one chylothorax, and one spinal cord deficit (the latter two in same patient). The complications in the pedicle screw group were: one dural lesion, one transient loss of MEPs, one implant failure, one pleural effusion, and one positive sagittal balance due to lumbosacral junctional kyphosis causing walking difficulties after instrumentation to L5. An L3 pedicle subtraction osteotomy and pelvic fixation was performed, which solved this issue. One patient experienced transient loss of MEPs during scoliosis surgery due to a high thoracic pedicle screw inserted into the spinal canal. After screw removal, MEPs returned and the patient recovered uneventfully without any neural deficits. There was one paraparesis in the hybrid group necessitating urgent re-decompression of the anterior spinal cord due to compression of the bone graft applied anteriorly after VCR with full recovery. The implant failures included one rod breakage just above the iliac connector and one iliac connector breakage. Altogether, one patient in both groups required revision surgery.

**Table 4:** Complications in the study groups

	Hybrid (*n* = 15)	Pedicle screw (*n* = 17)
Any complication (*n*)	6	5
Deep wound infection (*n*)	0	0
Implant failure (*n*)	1	1
Dural lesion (*n*)	0	1
Pneumo-/hemothorax (*n*)	1	1
Pneumonia (*n*)	1	0
Superior mesentery artery syndrome	1	0
Chylothorax	1	0
Urosepsis	1	0
MEP loss/transient neurologic deficit	1	1
Positive sagittal balance needing revision surgery	0	1

### Subgroup analysis

In a subgroup analysis, patients with nonneuromuscular scoliosis were excluded. Correction of the major curve was significantly better at 6 months (mean 60 vs. 73 %, *p* = 0.0020) and at 2-year follow-up in the pedicle screw group compared to the hybrid group (mean 58 vs. 72 %, *p* = 0.0059). The operative time tended to be shorter in the pedicle screw group than in the hybrid group (mean 7 h 40 min vs. 6 h 22 min, *p* = 0.074).

## Discussion

### Limitations of the study

The current study represents a comparative clinically based follow-up study in a consecutive series of patient groups undergoing surgery for severe scoliosis with either hybrid or total PSI. The data presented have been collected via a prospective systematic data collection system, although the study design was retrospective in nature. Selection between hybrid and total PSI, as well as between anteroposterior versus posterior-only surgery represents more development in the current surgical techniques, with a tendency to perform as much surgery via the posterior-only approach due to the pulmonary complications. Since the findings of this study represent a single-surgeon series, it is possible that the results somewhat reflect the learning curve of the senior surgeon during the study period. The etiology of scoliosis was mixed in this population. The higher implant density may be one of the explaining factors for enhanced deformity correction in the pedicle screw group as compared with the hybrid group. Pedicle screw patients had bilateral segmental fixation and also more Ponte osteotomies and pelvic fixations than the hybrid group. On the other hand, significantly more anterior discectomies were performed in the hybrid group to facilitate spinal deformity correction. These factors may also have influenced the better correction, less operative time, less blood loss, and more posterior surgery in the pedicle screw group compared to the hybrid group.

The position of the pedicle screws was not routinely checked with computed tomography (CT) scans. Thus, only complications caused by the malposition of pedicle screws can be reported in the present study. The question of when to include pelvic fixation in the correction of neuromuscular scoliosis remains somewhat controversial. More patients received pelvic fixation in the pedicle screw group as compared with the hybrid group. No patient was lost during the minimum 2-year follow-up.

### Comparison with previous findings

**Fig. 2:**
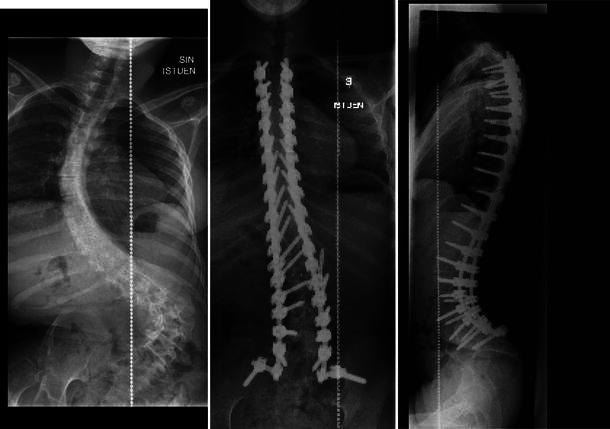
18-year-old girl with severe neuromuscular scoliosis. All posterior instrumentation using bilateral pedicle screws provided excellent correction and no loss of correction during the 2-year follow-up

Recently, there have been reports of better radiographic outcomes using PSI for adolescent idiopathic scoliosis as compared to hybrid constructs [3, 5, 6, 15]. Similar comparative studies in patients with severe scoliosis are lacking. Watanabe et al. [3] observed better correction of spinal deformities of 100° or more using pedicle screw than hybrid constructs. However, in their series, one-third of patients operated using pedicle screws had a VCR procedure, which obviously makes the comparison less reliable. In the present study, a similar number of patients in both groups underwent VCR, but, still, the correction of the deformities was better using PSI. Most of our patients had a neuromuscular scoliosis. In these patients, correction of the spinal deformity is less important than for patients with adolescent idiopathic scoliosis and, thus, the higher radiographic correction of the deformity when using pedicle screws is a positive finding, but may not be the most clinically relevant finding. On the other hand, correction of the severe neuromuscular scoliosis to allow for better seating, feeding, etc. is as important as the esthetics as the idiopathic curvature of less magnitude (Fig. 2).

Kuklo et al. [6] followed 20 patients with idiopathic scoliosis of 90° or more operated using PSI. Three patients had an anteroposterior approach. Spinal deformity correction was slightly less in their series (67 %) compared with the current series (73 %). Hamzaoglu et al. [8] reported a mean 51 % correction of severe idiopathic scoliosis in 15 consecutive patients who underwent wide posterior facet joint resections and PSI combined with intraoperative halo-femoral traction up 50 % of the body weight.

Koller et al. [9] recently reported on the treatment of severe scoliosis (mean 106°) with preoperative halo-gravity traction. One-third of 45 patients in this study underwent open anterior release, but none had VCR. The radiographic correction rate of 33 % was much lower than in the current series, but, also, the mean age of their patients was much higher (24 vs. 15 years in the current series).

The use of all PSI may also reduce the need for anterior surgery by providing better spinal deformity correction due to better biomechanical spinal instrumentation [15] and better control of crankshaft phenomenon in patients with open triradiate cartilage [20].

Major blood loss and associated coagulopathy are one of the major risk factors for severe complications and deaths in patients undergoing surgery for neuromuscular scoliosis [21]. Therefore, one of the major clinical advantages of pedicle screw constructs over hybrid or Luque–Galveston instrumentation seems to be limited blood loss. Passing a sublaminar wire or hook into the epidural space may start epidural venous bleeding, which can be difficult stop and can restart at every level of instrumentation. In contrast, the pedicle screw is an implant, which, when optimally placed, does not violate the spinal canal. If bleeding starts from the cancellous bone of the vertebral body, it usually stops after screw insertion. Additionally, while pedicle screws may sometimes take a longer time to insert than hybrid anchors, many anterior approaches were also avoided.

Bilateral top to bottom pedicle screw constructs are much more expensive than hybrid instrumentation. Whether the reduced number of anterior approaches, slightly shorter operative time, less blood loss, and better correction of spinal deformity justifies this construct can be debated.

A few studies have evaluated mortality in patients with severe scoliosis. Pehrsson et al. [1] observed increased mortality in untreated patients with scoliosis of 70° or more. Recently, Phillips et al. [16] reported a mortality rate of 18 % in patients with syndromic early-onset scoliosis. In the present study, patients with severe scoliosis of 90° or more were followed up to a mean of 5.5 years postoperatively to evaluate the mortality, which was only 3 %. All patients with severe scoliosis have a restrictive lung disease [2], which, according to studies in patients with idiopathic scoliosis, is only partially reversible even after major spinal deformity correction [4]. Additionally, most of our patients had a neurologic basic condition. Thus, the low mortality rate suggests relatively well balanced surgical indications, peri- and postoperative care, as well as a well-treated general health in these patients.

## Conclusions

The mortality rate was low up to a mean of 5 years follow-up. Pedicle screw instrumentation (PSI) provided shorter operative time, somewhat less blood loss, and better major curve correction with less need for anteroposterior surgery as compared with hybrid constructs in patients with severe scoliosis.
